# Yoga and meditation for menopausal symptoms in breast cancer survivors: a qualitative study exploring participants’ experiences

**DOI:** 10.1007/s00520-024-08603-2

**Published:** 2024-06-06

**Authors:** Mirela Bilc, Nina Pollmann, Analena Buchholz, Romy Lauche, Holger Cramer

**Affiliations:** 1 Institute of General Practice and Interprofessional Care, University Hospital Tübingen, Osianderstr. 5, 72076 Tübingen, Germany; 2 Robert Bosch Center for Integrative Medicine and Health, Bosch Health Campus, Stuttgart, Germany; 3 National Centre for Naturopathic Medicine, Southern Cross University, Lismore, NSW Australia

**Keywords:** Breast cancer, Yoga, Meditation, Menopausal symptoms, Qualitative study

## Abstract

**Purpose:**

Breast cancer survivors commonly experience menopausal symptoms, specifically when undergoing antihormonal therapy. Unfortunately, they often have a restricted range of treatment options available to alleviate menopausal symptoms. The objective of this qualitative study was to explore breast cancer survivors’ experiences and effects of a yoga and meditation intervention supplementing previously reported RCT outcomes.

**Methods:**

The qualitative data included in this study were part of a larger randomized controlled trial which evaluated the efficacy and safety of a 12-week yoga and meditation intervention on menopausal symptoms in breast cancer survivors. All participants who underwent the yoga intervention (*n* = 19) were invited to take part in semi-structured interviews after all quantitative data collection had been completed. Interviews (*n* = 9) were recorded, transcribed, and then coded into superordinate themes using thematic analysis.

**Results:**

Nine female participants were interviewed, and the following themes emerged: (1) representations and expectations from the yoga intervention; (2) course structure and implementation; (3) perceptions and effects of the intervention (at emotional, physical, behavioral, and spiritual level); (4) differences between the study yoga intervention and other physical activities.

**Conclusions:**

In accordance with the accounts of participants, yoga might offer a promising intervention for breast cancer survivors. All those interviewed either currently attended a yoga class or expressed a desire to continue practicing yoga. Additionally, our findings inform future studies regarding aspects such as the importance of extending outcome measures beyond specific cancer-related complains, the advantages of addressing homogenous groups (i.e., breast cancer specific), or considering that different intervention components might need different assistance to encourage long-term use.

**Supplementary Information:**

The online version contains supplementary material available at 10.1007/s00520-024-08603-2.

## Introduction

Breast cancer is the most common diagnosed cancer in females, with over 2.3 million cases in 2020, and it is projected to increase to about 3 million new cases by 2040 [1]. As a consequence of treatment progress and early detection, the number of breast cancer survivors has increased over time reaching an estimate of 7.8 million in 2020 (5-year prevalence) [1].

Menopausal symptoms pose a significant challenge for breast cancer survivors, arising either directly from cancer treatments like adjuvant endocrine therapy or ovarian function suppression in hormone-receptor-positive (HR +) breast cancer, or after discontinuation of hormone replacement therapy. In a recent systematic review [2], various symptom clusters associated with breast cancer were investigated across different stages of the disease. The menopausal cluster, evident both before and after cancer treatment, included symptoms such as hot flashes, vaginal dryness, night sweats, pain and joint discomfort, weight gain, mood swings, sleep disturbances, and difficulties with concentration.

Endocrine therapy to counteract estrogen-promoted tumor growth stands as the primary systemic treatment for HR + /ERBB2 − (human epidermal growth factor 2) breast cancer, affecting roughly 70% of patients [3]. Standard endocrine therapy typically entails daily oral antiestrogen medication for a span of 5 years. Treatment options vary depending on menopausal status, with tamoxifen proving effective for both pre- and postmenopausal women, while aromatase inhibitors (AIs) are effective only for postmenopausal women [3]. Both therapies commonly lead to hot flashes, with AIs additionally linked to other musculoskeletal symptoms such as joint pain and stiffness or muscle pain [3, 4]. These treatment-related issues not only impact quality of life but also raise concerns about adherence to endocrine therapies, thereby heightening the risk of recurrence for patients with poorly managed side effects [5]. While hormonal replacement therapy is the most effective means of addressing menopausal symptoms, it is contraindicated in breast cancer survivors due to the increased risk of cancer recurrence [6–8]. Recent clinical guidelines [9] and evidence-based approaches [10] provide comprehensive information on pharmacological, non-pharmacological, and complementary medicine treatment strategies for managing menopausal symptoms in women with a history of breast cancer. Yoga and relaxation techniques have been shown to mitigate hot flashes, with their use strongly recommended [9]. Additionally, the ASCO/SIO Pain Guidelines for Cancer Survivorship [11] suggest acupuncture and yoga for breast cancer survivors experiencing AI-related joint pain. The NCCN Guideline Survivorship Care for Cancer-Related Late and Long-Term effects [12] also recommends yoga as a non-drug treatment for hot flashes, arthralgias, and myalgias. Acupuncture is also endorsed for addressing vasomotor symptoms, arthralgia, and sleep disturbances [9].

Yoga is an ancient traditional spiritual practice rooted in Indian philosophy, whose modern practice commonly includes physical postures (asanas), breathing techniques (pranayama), and meditation (dhyana) [13]. Yoga has become a popular means to promote physical and mental well-being [14] and was shown to improve health-related quality of life as well as mental and physical health in breast cancer survivors [15, 16]. Yoga was also shown to have positive effects for female breast cancer survivors undergoing naturally occurring menopause [17, 18] as it is an effective mean of addressing the aggravating effect of stress on menopausal symptoms [19]. Empirical evidence shows that yoga reduces stress through increases in positive affect and self-compassion as well as inhibition of the posterior hypothalamus and decreases in cortisol [20].

A meta-analysis of 42 RCTs including yoga asanas versus active controls has shown that yoga was associated with reduced cortisol, systolic blood pressure, resting heart rate, and high-frequency heart rate variability, all physiological measures of stress [21]. Park et al. [22] have shown that changes in interoceptive awareness, mindfulness, and self-compassion but not self-control mirrored changes in stress during a 12-week yoga intervention.

However, the mechanisms by which yoga reduces menopausal symptoms in breast cancer survivors are relatively understudied.

To address this gap, we conducted in 2014 a randomized controlled trial (RCT), with the aim of investigating the effects of a 12-week traditional Hatha yoga intervention complemented with Buddhist meditation on menopausal symptoms in breast cancer survivors. Our results [23] showed that at week 12 the yoga group reported significantly lower total menopausal symptoms, less fatigue, and improved quality of life. These effects persisted at week 24, except for psychological menopausal symptoms. The short-term effects of our intervention were both clinically and statistically significant as 47.4% of the participants in the yoga group reported a reduction of at least 7 points on the Menopause Rating Scale total score compared to 9.5% in the usual care group. While RCTs are considered the gold standard to assess the effectiveness of an intervention, there are multiple ways in which they can benefit from the use of additional qualitative measures [24]. The present qualitative study aims to capture participants’ expectations and experiences regarding the yoga intervention beyond RCT-reported outcomes. This has the potential to uncover unmet needs, preferences, and priorities for yoga interventions that might not be apparent through other research methods. It could also help in advancing patient-centered care by promoting a more profound understanding of breast cancer survivors’ representations about yoga intervention.

## Methods

### Study design

This study utilized a qualitative research approach informed by interpretative phenomenological analysis (IPA). IPA, rooted in phenomenology, enables researchers to delve into participants’ distinctive viewpoints, offering valuable insights. The qualitative data analyzed in this study were part of a larger open-label, randomized controlled trial whose quantitative results have been previously reported [23, 25]. The analysis and publication of the qualitative results were delayed due to limitations in resources, specifically concerning funding and personnel availability. The present study includes data from the yoga intervention group, which entailed weekly, 90-min Hatha Yoga group practice complemented with meditation practices and approximately 30-min home practice over a period of 12 weeks. A complete list of postures, breathing, and meditation practices is available (see Online resource: Postures, breathing, and meditation practices).The study was approved by the Ethics Committee of the University of Duisburg-Essen (approval number 13–5421-BO) and was conducted in accordance with the ethical standards of the Declaration of Helsinki. Written informed consent was obtained prior to study participation.

### Participants

The RCT sample was recruited by the study physician at the Department of Gynecology Certified Breast Center at Malteser Hospital St. Anna.

All RCT participants from the yoga intervention group (*n* = 19), irrespective of the number of sessions completed, were invited to participate in the interviews at the end of the intervention. At this point, all quantitative data was completed. Nine out of the 19 participants agreed to participate in the semi-structured interviews. Reasons for non-participation included time constrains, lack of motivation, or lack of incentives. The mean age of participants in the yoga intervention group was 48.3 years, 31.6% were employed full-time, 52.6% were employed half-time, 5.3% were home keepers, and 10.5% were disabled. The mean reported time since surgery was 24.6 months, with more than half (*n* = 11) reporting stage I cancer, *n* = 7 stage II, and *n* = 1 stage III. Most women were receiving antiestrogen medication (*n* = 17) while none was receiving any nonhormonal treatment for menopausal symptoms. They attended a mean of 9.7 yoga classes and practiced on average 35.3-min yoga at home.

### Data collection

Each of the nine participants who consented to the interviews was provided with individual appointments tailored to their availability. The mean time between end of intervention and interview was 8 months (August–September 2014). The interviews took place at the Breast Center and were all conducted by a female, PhD-level psychologist (R.L.) who was trained and had prior experience in conducting qualitative studies. She had no background in psycho-oncology. There was no risk of power imbalance as the interviewer had no contact to the study participants before the interviews, nor afterwards. This ensures that participants felt comfortable and able to freely share their thoughts during the interview.

During these appointments, participants were invited into private rooms where they expressed informed consent to participate and to have their interviews pseudonymously recorded. An interview guideline was prepared in advance and used during interviews (see Online resource: Semi-structured interview guide). Interview questions were based on prior qualitative studies by our group on yoga in chronic pain [26] and colorectal cancer (unpublished results). The interviewer employed follow-up questions to further clarify and solidify the individual statements made by the participants. Participants were also given the opportunity to address aspects that they felt were particularly important and which may not have been previously addressed. Interviews were recorded using audio-only recording equipment and then manually transcribed verbatim by two medical students (A.B. and N.P.). Before transcription, they listened to the recording to familiarize themselves with the content. All personal identifiers were removed from the transcript to ensure participants’ confidentiality. A final quality check was performed. No field notes were taken.

### Data analysis

Thematic analysis, a method consistent with the foundational principles of IPA, was utilized for data analysis and the identification of themes. The transcribed text files were uploaded into the MAXQDA, which is a specialized software for qualitative data analysis which allows comprehensive data management. De-identified transcribed files were automatically imported into the software and then organized into a research project which could be accessed by all researchers involved in data analysis. Created codes were then organized into code groups, to reflect higher levels of abstraction. Before the first text analysis, two independent coders (A.B. and N.P.) discussed and decided upon a list of pre-set codes based on the interview guidelines and topics of interest. The following codes were included: complains, previous experiences, expectations, difficulties with the exercises, group aspect, yoga at home, perceived effects, continuing yoga. This served as orientation for the first text analysis, which encouraged inductive coding and thus further development of codes. A second text analysis took place with renewed assessment by the same two independent coders of the individual text passages and assignment of the adjusted codes. The independent codes were then merged into a single version after discussion and adjustment of discrepant classifications and a final crosscheck. Subsequently, these codes were combined into superordinate themes primarily by means of inductive category formation. These individual steps took place in regular exchange and discourse with members of the working group (H.C. and R.L.). No participant-checking was adopted.

## Results

### Themes

#### Representations and expectations from the yoga intervention

For some participants, the representation of yoga included the very conscious and slow execution of poses such as the sun salutation. For others, the image of “wacky” (P6) practitioners chanting Om while standing on their heads or sitting cross-legged was the predominant image. One participant describes the discrepancy between growing popularity and lack of knowledge as follows:

*On the one hand, yoga schools and yoga teachers are springing up like mushrooms [...], you can buy all this equipment again and it becomes more of a trend. On the other hand, there are still people who smile at it, partly, I think, out of ignorance, because they haven’t even looked into it.* (P9)

Some participants had already gained a more realistic insight into yoga practice through prior experiences in the context of medical rehabilitation, yoga classes at gyms, or previous contact with other relaxation techniques such as progressive muscle relaxation, Qigong, or Tai Chi.

Most participants had positive expectations regarding improvements in their general condition, symptom relief, and increased relaxation and mobility.

*I already had an idea that it would do me good, that it would bring me relaxation, that it would take me away from everyday life a bit, and that I would get a space where I could completely relax.* (P2)

On the other hand, there were also participants who were reserved, had no expectations at all, or were even skeptical. “*I didn’t necessarily wait for a positive effect*” (P3). In particular, those who described themselves as stress-prone or restless perceived yoga as a tool for stress management and relaxation. The idea of having the chance “*to do something for yourself*” (P3) was mentioned several times as the reason for participation in the study. Many participants have developed this attitude in the context of cancer and yoga, as a combination of physical and mental components was considered a successful way to implement this.

Viewing it through a diversity, equity, inclusion (DEI) lens, it is important to note that the participants interviewed did not discuss these dimensions concerning their involvement in yoga programs overall or specifically in relation to this study.

#### Course structure and implementation

Overall, participants liked the structure of the course and felt comfortable. Teaching the basic breathing techniques before incorporating the postures was considered a suitable sequence “*in order to first […] calm down*” (P2).

The concept of a group intervention was endorsed by most participants. Some found it pleasant or even a supportive factor. The latter was due to the fact that they all had the same condition and thus could more easily empathize with each other’s problems.

*The people all know what you have behind you and there is also consideration for it.* (P6)

Some participants, however, perceived the group rather neutral, focusing on themselves and paying less attention to others in the class. According to one participant, briefly introducing the patients in the first yoga session would have been beneficial.

The yoga teacher consistently received positive evaluations as she explicitly involved participants and inquired about undesirable effects but also positive changes. Participants thus felt that they were in good hands. They appreciated the detailed instructions for postures and the outlining of mechanisms of actions of single exercises, which contributed to the overall positive experience of the yoga course. Being able to observe the teacher and others during the class, as well as teachers’ feedback, fostered participants’ confidence. Therefore, many reported favoring in-person classes over practicing yoga on their own at home.

*She always took you with her.* S*he radiated an incredibly positive energy, I thought that was great. So, you always came out of it really inspired.* (P1)

*You are then also guided, and I also found that really positive about this yoga teacher, she did it incredibly well*. (P3)

Regarding the yoga components, the breathing exercises were partly perceived as requiring some practice before mastering the techniques, but at the same time very beneficial. Other practices, such as chanting or meditation, were perceived problematic initially, because the majority of participants had not experienced them before. Some participants were not used to the physical exertion and therefore experienced some difficulties such as muscle soreness and fatigue. Through regular exercise, participants were able to notice significant improvements: “*It got better from time to time. It became easier each time*” (P6). Initial physical limitations due to pre-existing conditions could be reduced by regular participation in the course.

*I did well [in yoga course]. I have some difficulties with my hands, and then I would only support myself on my hands for short periods of time, and then I would use my forearm or something, although it got a bit better over time. Or at the beginning, I couldn’t kneel down at all. And that was no longer an issue afterwards. Even now, I can still do it.* (P5)

While breathing exercises were widely integrated into everyday life, this was not the case with postures. Participants mentioned that it had been difficult to perform these without guidance and mentioned barriers such as insufficient space or distractors.

#### Perceptions and effects of the intervention

##### Emotional level

Many participants consistently reported “*good and positive thoughts*” (P2) associated with the yoga classes. In particular, the feeling of relaxation was mentioned as reaching a level at which they “*simply [were] completely relaxed […] and thought of nothing else*” (P2). The interplay of physical exertion and relaxation resulted in a pleasant feeling of fatigue:

*After the course I felt calmer and pleasantly tired. So, a fatigue that is so nice (P2); Exhausting yes, but it is incredibly pleasant in a different way.* (P8)

The increase in self-efficacy and stress resistance was an essential aspect in participants’ experience. Yoga helped them cope with feelings of vulnerability and fear of disease recurrence, by conveying positive ways of thinking. In addition, they felt empowered to contribute to the course of their health condition and no longer felt helpless. They became aware of the strength of their own body and that they could contribute to their own health.

*This course has at least given you the feeling that you can make your own contribution by simply dealing with yourself differently and saying, «So, I have a bit of control over this myself. My body can do a lot by itself and I can strengthen my immune system».* (P1)

##### Physical level

One change rated as particularly important was improved body awareness and perception.

*That you really have a left side and a right side. [...] I did not have these perceptions before. [Through] this tightening of the lower legs, the thighs, the chest [...], the perception was definitely strengthened.* (P6)

For most participants, the underlying attitude of paying more attention to their bodies had already developed following the cancer diagnosis. Yoga was an opportunity to address that, as it taught them to be more mindful and more responsive to signals from their bodies. This in turn helped identify symptoms like hot flashes early on and cope better. This strengthened perceived sense of control over the symptoms and led to a significant relief in daily life.

*So, before, these hot flashes just came, I couldn’t tell why. [...] and now I have the feeling [...] that they are [at least mostly] predictable for me. I know why I get them at that moment, and for me it’s usually when I get stressed, and through yoga I’ve learned to deal with stress a little bit better, and that’s why I think it can be reduced. I simply don’t let it get to the point where it happens in the first place.* (P1)

Yoga had “*brought back physical fitness*” (P8), observed as increased muscle strength, mobility, and improved physical condition. Overall, participants perceived their bodies as more resilient and capable, and everyday activities such as climbing stairs felt easier. For some participants, the course resulted in an improvement in posture. The body structure was strengthened and they were now more conscious of maintaining a healthy posture, which also led to a change in the way they were perceived by others. In the area of somatic complaints, many recorded an improvement in hot flashes, musculoskeletal complaints, and sleep difficulties. A decrease in pain, notably in the back and joints, as well as a reduction in joint stiffness, was observed. Issues stemming from prolonged hospitalization, such as muscle mass reduction, could also be alleviated. The majority of women reported a significant enhancement in sleep quality, particularly on the evenings following the yoga sessions. There was a considerable decrease in nocturnal awakenings, and the duration of time taken to fall asleep was also shortened. If they did wake up during the night, they often found it easier to return to sleep through the breathing and relaxation exercises.

*I slept through the night on the days when I had the [yoga] class, where I really had difficulties before and am having them again now. [...] I was a bit better physically. Climbing stairs was easier and everything, basically. [...] I was fitter.* (P6)

*Basically, the day I attend the yoga class, I sleep soundly through the night.* (P8)

Some participants questioned whether these improvements were due to increased physical activity in general and not specific due to yoga. Others, however, described that previously performed physical activities did not have such positive influence and that they could therefore clearly attribute these effects to yoga.

*I think I’m really doing something for my body [yoga], for the muscles and tendons and everything, but I would never get sore muscles after yoga […] So yes, you just feel really good without thinking you’ve burned calories and everything [..] your performance increases without having the feeling that you have been running around the lake for 1 ½ hours and totally exhausted yourself.* (P7)

Participants however emphasized the notion that, in order to maintain these effects, regular practice would be required. The persistence of the effect was evaluated differently depending on the complaint and the individual person. Improvement of sleep disturbances and relaxation lasted a few days after each class; the improvement of the musculoskeletal system mostly lasted until the next course and for some time after completion, as did the increased body awareness.

##### Behavioral level and everyday life

Participants reported that through better awareness of their own body and its needs, they learned to allow themselves more breaks without feeling guilty. Symptoms were no longer suppressed and the view that they had to keep going no matter what had “*completely changed*” (P2).

Participants reported frequently using breathing exercises in everyday life to cope with stressful situation. The breathing exercises allowed them to consciously slow down, relax, and refocus.

*Then I say to myself, «Come on, now stay calm for once, breathe into your stomach three times like this, and then think about it again calmly».* (P3)

Thanks to the newly gained tools in terms of stress and symptom management, participants felt a significant relief in their daily lives. A single participant described not having noticed any effects on her daily life. Without exception, each of the interviewed participants reported either already participating in another yoga class or having the desire to continue yoga.

##### Spiritual level

The spiritual level was more of a peripheral issue for most participants. The majority had an open attitude towards it and were partly curious; others found no connection to spirituality or were skeptical:

*In this particular case, [...] the therapist was also a Buddhist and that is a special constellation, which I don’t think you usually have with a yoga teacher. [...] I do believe that this made the yoga course different, because her whole attitude and her whole charisma is characterized by this, I think. That made the course even more special.* (P4)

*Well, maybe I can ground myself somehow, but that I would follow the spiritual so completely, no, I’m not for that now either.* (P7)

Some participants believed that access to spirituality only developed in the course of a long-term yoga practice. Participants reported that the authenticity of the yoga class was strengthened by the Buddhist faith of the teacher.

#### Differences between the study yoga intervention, previous yoga experiences, and other physical activities

Participants mentioned differences between the yoga intervention in the study and other yoga courses, such as those in fitness centers. In the latter case, new participants, with less knowledge and experience, join already existing groups. This makes it difficult to learn the basics and, as one participant said, made them feel like a “*job newcomer*” (P8). This was not the case during the study, as everyone started the course at the same time. Addressing yoga philosophy and its potential effects, the “*mental level*” (P1) was also neglected in most fitness centers, in contrast to the yoga intervention in our study.

The comparison with other physical activities was also often mentioned. Participants reported that during yoga they respected their own limits more, were less exhausted, and placed more emphasis on precision and execution. The physical effort during yoga would also cause sore muscles especially in the early phases, but at the same time participants would experience a state of relaxation. One participant did not perceive yoga to be much different from other types of physical activity.

In addition, some participants expressed their belief that it would be beneficial to have regular yoga classes offered in the hospital. It was often mentioned that it might be particularly helpful in the early stages of the disease:

*I could imagine that it would also help many women, if one were to do this already at the beginning of the disease. If only for relaxation, because [...] many have fallen into quite a hole at first, and that would perhaps distract and help a bit if you did this [yoga]* (P2);

*But I think, especially if you have gone through such a serious illness like cancer, then I would find that very important [...] and very positive to learn something like this [yoga]. I would have liked to have that in rehab, for example, that I would have such an opportunity to learn something like this.* (P4)

## Discussions

The present study provides a unique perspective into breast cancer survivors’ expectations and experiences related to a yoga intervention which supplements findings from randomized controlled trials [23, 25].

### Expectations and course format

While representations of yoga and previous experiences varied among participants, the attitude was primarily a positive one with participants being open and curios. This is of fundamental importance for recruiting into yoga and yoga trials, however does not automatically translate in high expectations regarding the effectiveness of the intervention. As seen in our analysis, expectations ranged from being skeptic about yoga to anticipation of specific symptom improvement.

Similar to other exercise interventions [27, 28], the majority of female breast cancer survivors reported benefitting from the group intervention and preferring it over individual home practice. Having all group members share the same disease and start the intervention at the same time as well as offering a comprehensive course which included also yoga philosophy seemed to positively distinguish our intervention from those offered elsewhere, such as fitness studios. Explanations regarding the effects of specific exercises and the flexibility of adjusting them to participants’ own capabilities were additional factors that contributed to participants’ satisfaction with the intervention.

### Effects of the intervention

Yoga was reported to have contributed to an increased body awareness and self-efficacy. This is in line with previous reports of improvements in body awareness [29–32] and self-efficacy[33–35] following yoga, as well as the potential moderator effect of body awareness in the relation between yoga practice and well-being [36] and its centrality to mind–body therapies in general [37]. This double effect allowed participants to recognize stressful situations as triggers for hot flashes, while also providing an effective means to counteract the effects of stress. The need for finding coping strategies when faced with the probability of cancer remission seems to be particularly salient among this group of participants and it was also mentioned among reasons for participating in the study. Future studies should explore yoga’s potential, especially in line with findings showing that the use of active-adaptive coping strategies is significantly associated with post-traumatic growth in female breast cancer survivors [38]. Furthermore, mentions were made in some of the interviews regarding offering yoga courses early on in the disease management plan, which is supported by evidence showing positive effects of yoga in female breast cancer survivors during chemotherapy [39, 40].

Regarding the comparison with other physical activities, the majority of the interviewed participants highlighted the advantages of yoga over other forms of exercise. Alongside an increase in physical fitness, yoga also improved relaxation, which might be particularly beneficial in helping cancer patients overcome barriers such as fatigue and discomfort, typically associated with low adherence to exercise interventions [41].

### Practice beyond the study

Our analysis also shows that among yoga components, breathing exercises were more likely to be used in everyday life, particularly in the context of stress management. This can be partially attributed to the lack of space and equipment necessary for yoga postures, while breathing is easily accessible also in public situations. Future studies or yoga courses could integrate this knowledge and help patients overcome fears of home injuries by addressing them in class as well as providing participants with additional detailed materials to encourage home use. Beyond the practical aspects, these results might also underline the importance and usefulness of breathing as a component of the yoga intervention. This is supported by a recent systematic review on the effects of yogic breathing which showed improvements in cancer-related fatigue, emotional symptoms, sleep, and quality of life for cancer patients [42]. Furthermore, it is encouraging to see that after attending the intervention, female participants felt empowered and adjusted what they have learned in the course to fit their own needs.

### Limitations

This study has some limitations regarding age and incompleteness of the data, sample size, and depth of the interviews. Resource constrains led to a significant delay in analyzing and publishing these qualitative findings, subsequent to the publication of the RCT results. Given the evolving perspectives and understanding of social phenomena over time, it is plausible that the acceptance and use of yoga differed at the time the interviews were conducted compared to the present state. Furthermore, this delay posed challenges in data management, resulting in the inability to match interview data with participant demographics. This limits the contextual understanding and diminishes the transferability of our findings. Since the interviews were conducted with participants who have completed the yoga intervention, we were limited in terms of exploring potential drop-out reasons. It might be particularly informative for future studies to include qualitative analysis accounting for study discontinuation. Also, we cannot exclude a self-selection bias since despite our efforts to encourage participants with no or limited benefits to attend the interviews, those benefitting from the intervention were more likely to participate. Nevertheless, reports of no observed changes were also mentioned in the interviews.

## Conclusion

In conclusion, this study provides valuable insights regarding yoga interventions for breast cancer survivors. It has drawn attention to the importance of assessing outcomes beyond cancer-related complains, such as overall fitness and self-efficacy, and suggests that female breast cancer survivors might benefit more from “custom-made” interventions targeting problems specific to this group over generic ones.

### Supplementary Information

Below is the link to the electronic supplementary material.

Supplementary file1 (PDF 79 KB)

Supplementary file2 (PDF 96 KB)

## Data Availability

The data that support the findings of this study are available from the corresponding author, H.C., upon reasonable request.
