# Supplementary material for: Yoga and meditation for menopausal symptoms in breast cancer survivors: a qualitative study exploring participants’ experiences
Source: Support Care Cancer. 2024 Jun 6;32(7):413. doi: 10.1007/s00520-024-08603-2 (PMC11156729; doi:10.1007/s00520-024-08603-2)
Supplement: Supplementary file 1 — Supplementary file1 (PDF 79 KB) [file 520_2024_8603_MOESM1_ESM.pdf]

Article title:

Yoga and meditation for menopausal symptoms in breast cancer survivors: a qualitative study exploring participants' experiences

Journal:

Supportive Care in Cancer

Authors:

Mirela Bilc,[1,2] Nina Pollmann,[1,2] Analena Buchholz,[1,2] Romy Lauche,[3] Holger Cramer[1,2,3]

[1]Institute of General Practice and Interprofessional Care, University Hospital Tübingen, Tübingen, Germany.

[2]Robert Bosch Center for Integrative Medicine and Health, Bosch Health Campus, Stuttgart, Germany.

[3]National Centre for Naturopathic Medicine, Southern Cross University, Lismore, NSW, Australia.

Corresponding author:

Holger Cramer, Institute of General Practice and Interprofessional Care, University Hospital Tübingen, Osianderstr. 5, 72076 Tübingen, Germany (Holger.Cramer@med.uni-tuebingen.de)

Online resource: Postures, breathing and meditation practices

Yoga Postures (Asana)

- Child pose (Balasana)[a]
- Cobra pose (Bhujangasana)[a]
- Corpse pose (Shavasana)[a]
- Crocodile pose (Makarasana)[a]
- Fish pose (Matsyasana)[a]
- Forward bend (Paschimothanasana)[a]
- Half bridge pose (Setu Bandhasana)[a]
- Half twist pose (Ardha Matsyendrasana)[a]
- Shoulder stand (Sarvangasana)[a]
- Staff pose (Dandasana)[a]
- Sun salutation (Surya Namaskara)[a]

Breathing Techniques (Pranayama)

- Alternate Nostril Breathing (Anuloma viloma)[a]
- Diaphragmatic breathing[a]
- Kapalabhati[a]
- Vase breathing[b]

Meditation Techniques (Dhyana)

- Body scan[b]
- Calm abiding meditation (Shine)[b]
- Mantra meditation[a,b]
- White Tara meditation[b]

[a] Derived from traditional Hatha yoga
[b] Derived from Buddhist meditation
