# Supplementary material for: Yoga and meditation for menopausal symptoms in breast cancer survivors: a qualitative study exploring participants’ experiences
Source: Support Care Cancer. 2024 Jun 6;32(7):413. doi: 10.1007/s00520-024-08603-2 (PMC11156729; doi:10.1007/s00520-024-08603-2)
Supplement: Supplementary file 2 — Supplementary file2 (PDF 96 KB) [file 520_2024_8603_MOESM2_ESM.pdf]

Article title:

Yoga and meditation for menopausal symptoms in breast cancer survivors: a qualitative study exploring participants' experiences

Journal:

Supportive Care in Cancer

Authors:

Mirela Bilc,[1,2] Nina Pollmann,[1,2] Analena Buchholz,[1,2] Romy Lauche,[3] Holger Cramer[1,2,3]

[1]Institute of General Practice and Interprofessional Care, University Hospital Tübingen, Tübingen, Germany.

[2]Robert Bosch Center for Integrative Medicine and Health, Bosch Health Campus, Stuttgart, Germany.

[3]National Centre for Naturopathic Medicine, Southern Cross University, Lismore, NSW, Australia.

Corresponding author:

Holger Cramer, Institute of General Practice and Interprofessional Care, University Hospital Tübingen, Osianderstr. 5, 72076 Tübingen, Germany (Holger.Cramer@med.uni-tuebingen.de)

Online resource: Semi-structured interview guide
(Original in German, translated into English for publication)

1. Welcome and introduction

This is an interview about the study "Randomized controlled trial of the effect of yoga on menopausal symptoms in patients with breast cancer". The interview will focus on your experience of the yoga course and how you are currently feeling. The interview is expected to last no more than 45 minutes. The conversation will be tape-recorded, later transcribed and anonymized - i.e. all names, places and statements that could allow a connection to your person will be changed. Your statements will, of course, be treated as strictly confidential. Do you have any questions? Then let us get started with the interview.

2. Narrative prompt

You have recently participated in a yoga course as part of the study. I would therefore like to ask you about how you have experienced the yoga course, as well as how you are currently feeling. There are no right or wrong answers, I am interested in everything that is important for you.

Expectations regarding the yoga course	*Possible follow-up questions*
Please think back to the time before the yoga course. What were your expectations and/or fears about the course at that time?	To what extent have you had contact with yoga before?
What made you decide to join the yoga course at that time?	Why did you decide to attend the course?
	To what extent did you decide to attend the course because of existing medical conditions?
At that time, how high did you consider the possibility of yoga to influence your well-being?	

Experiencing the yoga course	*Possible follow-up questions*
How did you feel about the yoga course in general?	What did you find good? (What did you find particularly good)
	What didn't you like so much? (Did something bother you?)
	Can you describe how you felt in the yoga class?

	To what extent did you notice differences before and after the yoga course?
	To what extent was the course as you imagined it?
When you think about the individual elements of the yoga class: which ones appealed to you the most and which ones were difficult for you?	What role did the physical exercises play for you?
	To what extent were some of the physical exercises difficult for you?
	How did you feel about the sun salutation?
	How did you perceive the breathing exercises?
	How did you feel about singing the OM?
	How did you experience the meditation at the end?
	Which meaning had the meditation for you?
	What role did the physical exercises play for you?
What was it like for you to practice yoga in a group with other patients?	How did you experience the dynamics in the group?
	To what extent did the group influence how you practiced yoga?
Can you describe what motivated you to attend yoga classes regularly?	
How often did you practice alone at home?<br>What was your experience like?	To what extent do you think the frequency of practice was important?
To what extent were there circumstances that favored participation in the course?	
To what extent were there circumstances that made it difficult or impossible to participate in the course?	To what extent did your symptoms hinder or prevent you from participating?

Symptoms/everyday life/attitudes/comparison	*Possible follow-up questions*
How do you assess the possibility to influence your well-being through yoga today?	To what extent do you currently have any symptom complaints?
	What impact do you think yoga has had on your symptoms?
	In what ways might yoga have influenced your symptoms?
To what extent has your everyday life changed since you started the yoga course?	Before starting the yoga class, to what extent did your symptoms limited your daily activities or social interactions?
	How has yoga practice influenced these limitations?
To what extent has your attitude about your body changed since you started yoga classes?	To what extent have the feelings about body changed since you began yoga classes?
To what extent has your attitude toward cancer and its associated symptoms changed since you began yoga classes?	To what extent have your feelings related to cancer changed since you started the yoga class?
	To what extent does your cancer burden you today?
	Has the yoga course impacted how you deal with cancer?

Body awareness comparison	*Possible follow-up questions*
Has your body awareness changed since you started yoga classes?	What differences in your body awareness have you noticed since starting yoga classes?
	How exactly would you describe these changes?
	When did you first notice these changes?
To what extent can you make a connection between yoga practice and your body awareness?	What aspects of yoga have an impact on your body awareness?
	Do you see other connections between your lifestyle and how you perceive your body?

How important is body awareness for you today?	What do you pay attention to the most?

Lifestyle (social environment)	*Possible follow-up questions*
When you look at your current lifestyle, what is most important to you?	Did the yoga course have an impact on how you live your life?
	How has your private environment reacted to the fact that you practice yoga? Did you feel supported?
	Have you noticed any changes regarding your diet?

Spirituality and religion	*Possible follow-up questions*
Did you perceive dimensions in the yoga class, that went beyond the purely physical?	(Can you describe this in more detail?)
	(How would you describe 'this area'?)
	(What does this mean to you?)
	In your opinion, are there differences between yoga and other sports?
Yoga is often associated with spirituality, have you had any experiences in this regard?	Were these experiences new to you, or do you already know them from other contexts?
	Did these experiences have an impact on your everyday life?
Was there a connection for you between yoga and your faith?	
When or where do you feel most in tune with the cosmos, or part of something bigger?	
How important is 'this area' (faith, religion, spirituality) we just talked about for your life?	

Concluding questions, current feelings
(Finally, can you tell me briefly in summary what you think about yoga?)

Thank you very much for your willingness to participate in this interview. Would you like to add anything else that would be important to you regarding the yoga course, which we have not gone into so far? (...)
Thank you again for your participation.
